# Peptide-Based Vaccinology: Experimental and Computational Approaches to Target Hypervariable Viruses through the Fine Characterization of Protective Epitopes Recognized by Monoclonal Antibodies and the Identification of T-Cell-Activating Peptides

**DOI:** 10.1155/2013/521231

**Published:** 2013-06-26

**Authors:** Matteo Castelli, Francesca Cappelletti, Roberta Antonia Diotti, Giuseppe Sautto, Elena Criscuolo, Matteo Dal Peraro, Nicola Clementi

**Affiliations:** ^1^Microbiology and Virology Institute, Vita-Salute San Raffaele University, 20132 Milan, Italy; ^2^Laboratory for Biomolecular Modeling, Institute of Bioingeneering, School of Life Sciences, Ecole Polytechnique Fédérale, 1015 Lausanne, Switzerland

## Abstract

Defining immunogenic domains of viral proteins capable of eliciting a protective immune response is crucial in the development of novel epitope-based prophylactic strategies. This is particularly important for the selective targeting of conserved regions shared among hypervariable viruses. Studying postinfection and postimmunization sera, as well as cloning and characterization of monoclonal antibodies (mAbs), still represents the best approach to identify protective epitopes. In particular, a protective mAb directed against conserved regions can play a key role in immunogen design and in human therapy as well. Experimental approaches aiming to characterize protective mAb epitopes or to identify T-cell-activating peptides are often burdened by technical limitations and can require long time to be correctly addressed. Thus, in the last decade many epitope predictive algorithms have been developed. These algorithms are continually evolving, and their use to address the empirical research is widely increasing. Here, we review several strategies based on experimental techniques alone or addressed by *in silico* analysis that are frequently used to predict immunogens to be included in novel epitope-based vaccine approaches. We will list the main strategies aiming to design a new vaccine preparation conferring the protection of a neutralizing mAb combined with an effective cell-mediated response.

## 1. Introduction

The development of vaccines directed against clinical relevant viral pathogens is perhaps the most important contribution of immunology to public health. Traditional vaccine preparations are based on attenuated or inactivated whole viruses or partially purified viral proteins. These strategies, although effective against a large number of pathogens, present drawbacks due to viral intrinsic characteristics such as poor or null *in vitro* replication and antigenic hypervariability [1].

In order to overcome these issues, quite a number of novel approaches have been developed, one of the most promising focusing on epitope-based vaccine preparation.

The possibility to use minimal structures such as peptides, or a mixture of them, as the main constituent of a vaccinal preparation, presents many advantages. Firstly, peptides can be easily produced *in vitro* reducing production costs and simplifying large-scale vaccine production procedures. Moreover, expression of peptides belonging to viral proteins does not necessarily require *in vitro* pathogens growth, overcoming viral culturing issues.

This strategy also presents safety benefits, zeroing problematic related to back mutations for attenuated viruses and reducing side effects due to possible improper immune response against viral antigenic determinants. 

Perhaps the most important aspect of using well-characterized synthetic peptides as immunogens is related to the specific triggering of both humoral and cell-mediated immune responses against a fundamental domain of a viral protein. Moreover, the possibility to remove antigen (Ag) domains activating suppressor mechanisms may elicit only a protective response targeting conserved functional regions shared among hypervariable viruses [2].

Despite these advantages, to date no epitope-based vaccines have been used in clinical practice. This is mainly due to low immunogenicity and difficulties related to the fine identification of protective epitopes and/or properly folded antigen structural motifs to be included in a vaccinal preparation. The latter is fundamental to properly activate an effective immune response. Furthermore, a main goal for a successful epitope-based vaccine approach is the identification of epitopes capable of eliciting both humoral and cell-mediated responses [3, 4].

**Figure 1:**
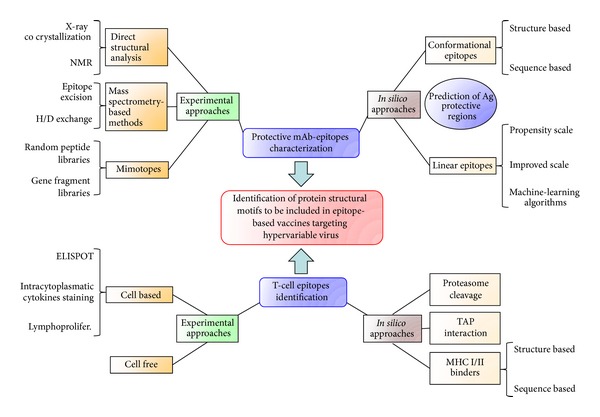
The described approaches to characterize protein structural motifs to be included in new vaccines targeting hypervariable viruses. The synergistic use of techniques combining experimental and *in silico* approaches is also shown.

Different strategies, spanning from antigen presentation techniques to *in silico* design of structural motifs to be included in vaccinal preparations, have been developed in order to overcome these issues. In this paper we review the most promising approaches in peptide-based vaccine setup applicable to hypervariable viruses. In particular we will focus on the methods at the interface between experimental and computational procedures aiming at the prediction of B and T-cell-activating peptides (Figure 1).

## 2. Selection of B-Cell-Activating Peptides: Immune Humoral Response as a Probe to Identify Crucial Domains

A crucial step in epitope-based vaccine design is the identification of antigens capable of eliciting a protective immune response specific for a pathogen of interest. Depending on the characteristics of the virus to be targeted, humoral and cellular response changes in relevance. As an example, the former plays a crucial role in conferring specific immunity for influenza virus infection. Many researches have been focused on the characterization of protective monoclonal antibodies (mAbs) targeting widely conserved hemagglutinin (HA) regions among different influenza subtypes [5–12]. Considering the clinical potential of mAbs endowed with such peculiar cross-neutralizing activity, their epitope characterization represents a valuable tool to identify functional and conserved epitopes potentially useful in an epitope-based vaccinal strategy.

Different methods, either exclusively based on experimental approaches or involving the use of *in silico* studies, have been applied to identify regions featuring the aforementioned characteristics. Several of the most frequently used methods are listed and discussed in the following.

### 2.1. Direct Structural Analysis of mAb/Antigen Complex

Structural resolution of a specific mAb in complex with its target through X-ray crystallography or nuclear magnetic resonance (NMR) is to date the only procedure to obtain interaction information at atomic level [9, 13]. However, considering methods complexity and inability to be applied to certain complexes together with low throughput features, X-ray crystallography and NMR represent useful tools to fully characterize the epitope of a single mAb but are not suitable for mapping all antigenic determinants.

### 2.2. Mass Spectrometry- (MS-) Based Techniques

The MS based techniques permit to define mAb epitopes at a medium resolution. All the MS-approaches aim at the identification of mAb footprint on the targeted antigen [14, 15]. Different experimental methods involving MS are widely described in the scientific literature. These approaches are mainly based on the protection of mAb binding site on the whole antigen from proteolytic digestion or protein modification (i.e., acetylation or deuterium incorporation), through its bond with the mAb itself [16, 17]. mAb-interacting fragments are subsequently identified through MS and mapped *in silico *on the whole antigen to define epitope sequence and structure. In particular, the computational analysis is generally performed excluding the “nonepitope” antigen regions (Ag unbound regions) followed by the mapping of Ag amino acid residues derived from MS analysis (e.g., not subjected to proteolytic digestions or deuterium incorporation) on the Ag crystal structure.

### 2.3. Mimotopes

Mimotopes are small peptides able to mimic antigenic conformational structures recognized by an antibody (Ab) paratope. The most frequently used approach to isolate specific mimotopes recognized by a mAb is based on the screening of a random peptide phage display through biopanning techniques [18, 19]. Alternatively, if the antigenic protein can be cloned and expressed from recombinant cDNA, a library composed by antigen fragments can be created and screened for positive binding to mAbs.

Selected peptides are then sequenced, aligned to antigen sequence, and, if available, superimposed to its three-dimensional (3D) structure, allowing the identification of the immunogenic domain. This process often requires the use of specific *in silico* tools, as epitope localization on antigen surface from mimotopes sequences might not be trivial; specific algorithms such as Mimox (http://immunet.cn/mimox/), Pepitope (http://pepitope.tau.ac.il/), and MimoPro (http://informatics.nenu.edu.cn/MimoPro/) are available online [20–22]. They all perform an alignment of provided mimotope sequences to a given PDB structure, returning epitope localization; identification can be done either on a single mimotope sequence or clustering all positive sequences and searching for a consensus patch on the structure. An online database named MimoDB 2.0 (http://immunet.cn/mimodb/) is also available online; it collects from the scientific literature thousands of mimotopes identified from random libraries providing information about identification methods, libraries, and respective protein [23].

Identification of mimotopes is a powerful technique as it easily allows to map many antigenic determinants at the same time using a polyclonal serum or to identify a single mAb epitope at a medium resolution [24, 25]. The canonical 18 mer peptides allow the study of conformational epitopes, as they are long enough to fold into a specific secondary structure. Moreover, it can be efficiently used when antigens 3D structure is not available, returning possible peptides to be used in a peptide-based vaccinal approach disregarding their structure.

### 2.4. *In Silico* Prediction of Linear Epitopes: Propensity Scale, Improved Propensity Scale, and Machine-Learning Algorithms

Continuous epitopes include ~10% of all known antibodies epitopes; while they comprise a minority of all epitopes found in nature, many computational methods focus on their mapping [26, 27].

Sequence-based algorithms represent the first attempt to predict B-cell epitopes located on a protein surface without *a priori* immunological data. Most of these algorithms, namely, *propensity scale *(or *amino acid scale-based*) methods, rely upon residues chemical and physical properties based on empirical data (i.e., hydrophilicity, flexibility, solvent accessibility, polarity, and presence of *β*-turns). Five of the most used amino acid scale-based methods are implemented at the Immune Epitope Database (IEDB) website (http://tools.immuneepitope.org/main/html/bcell_tools.html) [28]. A standard score to evaluate the performance of these methods is the *A*_ROC_ (Area under the Receiver Operating Curve) value. This value spans from 0 to 1 where a value of 0.5 matches with a random prediction, and 1 represents the ideal performance [29]. None of the methods implemented in IEDB website and listed previously exceeded the *A*_ROC_ threshold of 0.6 when benchmarked with three standard datasets, pointing out their low reliability in predicting linear epitopes. Only a small improvement in comparison with a random prediction is in fact demonstrated for single propensity scales [30].

Considering the amino acid scale-based methods as a starting point, novel algorithms combining different propensity scales and machine-learning methods have been developed. While the former strategy did not lead to substantial improvements, machine-learning methods have proven their efficacy when tested, exceeding the *A*_ROC_ threshold value of 0.6. The first generation of these hybrid algorithms comprises, among the others, ABCpred (http://www.imtech.res.in/raghava/abcpred/), a recurrent artificial neural network- (ANN-) based algorithm, and BepiPred (http://www.cbs.dtu.dk/services/BepiPred/), which combines a machine-learning method such as the hidden Markov model (HMM) with two propensity scale methods taking into account Parker's hydrophilicity and Levitt's secondary structure scales [31–34].

In the last few years several machine-learning algorithms exploiting Support Vector Machine (SVM) have been implemented as well, leading to a progressive prediction improvement in terms of accuracy, sensitivity, and specificity [35, 36]. 

Recently Lin et al. developed the algorithm BEEPro, an SVM-based learning-machine which uses fourteen physiochemical scales to generate a hybrid propensity scale including antigenicity, hydrophilicity, flexibility, composition, volume, charge transfer and donor capability, hydrogen bond donor capability, and secondary structure features. It is then further combined with an amino acid ratio propensity scale representative of the propensity of each amino acid to be part of an epitope and a position specific scoring matrix (PSSM) which reflects the evolutionary information of a peptide [37].

Considering these parameters, BEEPro, has been trained with the Sollner dataset comprising many non-redundant linear epitopes and proved itself to efficiently predict both linear and conformational epitopes, outperforming other prediction algorithms [38].

### 2.5. *In Silico* Prediction of Conformational Epitopes: Structure- and Sequence-Based Algorithms

Conformational epitopes mapping represents a challenging goal in different biological and medical fields. In the last few years many algorithms capable of predicting conformational epitopes have been developed. They can be divided in structure-based and sequence-based algorithms.

Structure-based algorithms work on three-dimensional (3D) proteins structure obtained either through X-ray crystallography or NMR and exploit different spatial parameters as well as amino acids statistics. CEP [39], together with DiscoTope (http://www.cbs.dtu.dk/services/DiscoTope/), is the first web server developed to predict both linear and conformational epitopes; it relies on residues solvent accessibility and defines a linear epitope when at least three consecutive residues satisfy the solvent exposure parameter. Conformational epitopes are then predicted considering linear epitopes whose C*α* is closer than 6 Å [39].

DiscoTope is a method oriented to conformational epitopes prediction; the algorithm bases its prediction on the combination of hydrophilicity, amino acids propensity score taken from a dataset of resolved antibody/antigen structures, residues spatial neighborhood, and area of relative solvent accessibility [40]. The 2.0 version of DiscoTope recently implemented includes novel strategies to define the spatial neighborhood and a half-sphere exposure to calculate surface exposure; it has been shown to outperform the majority of previous prediction algorithms [41].

After CEP and DiscoTope, many others machine-learning methods to predict conformational epitopes starting from a 3D structure have been developed; PEPITO (http://pepito.proteomics.ics.uci.edu/), SEPPA (http://lifecenter.sgst.cn/seppa/), EPCES (http://sysbio.unl.edu/EPCES/), and its improved version EPSVR (http://sysbio.unl.edu/EPSVR/) analyze 3D structures and aim at the division of antigens surface in epitopic and nonepitopic patches on the basis of different propensity scores and solvent accessibility; they all rely on training datasets comprising resolved antibody/antigen complexes [42–45].

Moreover, new algorithms try to improve analysis and broaden targets using linear sequences when structures are not available. ElliPro (http://tools.iedb.org/tools/ElliPro/iedb_input) can model proteins of unknown structure aligning their sequence in BLAST and then modeling structures with MODELLER; epitopes search is then performed approximating protein shape to an ellipsoid, calculating every residue protrusion index (PI) and finally clustering neighboring residues based on their PI values [46, 47]. As well as ElliPro, Epitopia (http://epitopia.tau.ac.il/) allows the user to input either antigen structure or sequence; the prediction algorithm calculates an immunogenicity score for each residue through a trained naïve Bayes classifier and clusters them, outputting a probabilistic score for each patch [48].

Despite the effort, none of the structure-based methods reached a high efficiency in terms of accuracy, sensitivity, and specificity. Unsuccessful attempts might be due to many aspects; first of all, the number of antibody/antigen resolved structures is too small to provide a robust statistical sampling of all possible epitopic patches. Moreover, datasets are affected by the low resolution of some structures. Another issue is the lack of consideration of proteins as complexes *in vivo*; during algorithms training, protein patches that are physiologically buried in protein-protein complexes can wrongly be considered as possible epitopes. Other problems come from the definition of an epitope in terms of which residues should be considered as part of it; this involves both the proximity threshold of surface residues to be used and the lack of consideration for buried residues below the epitopic patch. Finally, experimentally not all the possible epitopes of an antigen might have been identified. All these aspects lead to a biased training of the machine-learning algorithms, which in turn cause a prediction far from optimal [49].

Considering efficiency issues and limited available antigens structure, novel sequence-based methods have been developed. The first attempt is represented by the CBTOPE (http://www.imtech.res.in/raghava/cbtope/) algorithm, which reached better results than all structure-based algorithms. A SVM was trained with protein chains belonging to antibodies epitope; each residue was classified as binding or nonbinding and characterized to define residue-specific physiochemical and composition profiles. This strategy allows to define specific epitopic and non-epitopic patterns that are then applied to the local amino acid composition of the antigen; prediction is thus performed without considering the whole protein sequence but searching for epitopic patterns [50].

Recently two more sequence-based algorithms, the aforementioned BEEPro, and the method published by Zhang et al. outperformed CBTOPE results. Results succeeded by these three algorithms are related to the usage, besides many physiochemical properties, of matrices that try to identify specific nonlinear patterns for epitopic and non-epitopic patches.

Considering results achieved by CBTOPE, Zhang et al. tried to explore more potentially relevant sequence-derived features effective for the conformational epitopes prediction. Besides physiochemical characteristics and amino acids propensity to be part of an epitope, residues side chains have been clustered in thirteen classes to compute the propensity for each of them; moreover, a PSSM has been used as in BEEPro to calculate evolutionary conservation. A term representing the secondary structure is included as well. The random forest machine-learning algorithm is then used to classify each query protein patch on the basis of every feature creating an output ensamble and then rank the results. It is interesting to notice that Zhang et al. determined the PSSM to be the most effective feature in predicting epitopes explaining BEEPro performance [37, 49]. CBTOPE, BEEPro and the web server developed by Zhang et al. can provide a satisfactory output that can be used as a good starting point for further experimental evaluation confirming putative epitopes.

## 3. Identification of T-Cell-Activating Peptides

While moving towards an epitope-based vaccine strategy, both humoral and cell-mediated response have to be taken into account (Figure 1). An effective immunity has indeed to be mediated by the induction of neutralizing antibodies together with the activation of specific cytotoxic CD8 and helper CD4 T lymphocytes. Therefore, as well as with B epitopes, a great effort has been put in the characterization of peptides binding to major histocompatibility complex (MHC) of class I and class II that can be presented to TCRs and in their prediction from antigen sequence/structure [51, 52]. Many experimental techniques involving either cellular of biochemical assays have been developed, but complexity and costs of these methods address the need of reliable *in silico* approaches to reduce and guide them.

Protective T epitopes characterization involves different issues that are related to the complexity of their processing and presentation on MHC I and MHC II; merely screening all possible MHC-binding peptides does not in fact directly correlate to their role in inducing immunity. Physiological pathogen-specific T-cell activation involves in fact several steps, comprising antigen digestion by the proteasome/immunoproteasome, interaction with the transporter associated with antigen processing (TAP) protein for MHC I binding, binding to MHC and TCR recognitions. Efficient T epitopes prediction has to take into account all these aspects; ideal immunogenic peptides thus must be efficiently processed by the immunoproteasome and delivered by TAP into the endoplasmic reticulum to bind to MHC I. Moreover, considering the human leukocyte antigen (HLA) allelic diversity, effective vaccine peptides have to be recognized by haplotypes widely shared among the population [53, 54].

To date many online tools are available to predict cleavage, TAP translocation, and HLA specificity for MHC I and MHC II binding. Several databases reporting binding peptides are available online as well. The synergistic use of these tools can noticeably restrict the number of peptides to be experimentally analyzed. Here we describe *in silico* and *in vitro *approaches, reviewing the most used databases together with structure- and sequence-based prediction methods and experimental procedures used to validate algorithms output.

### 3.1. *In Silico* Approaches: Databases

As described previously, protective T epitopes prediction has to take into account different aspects.

A first analysis can be easily done using databases of well-characterized peptides recognized by T cells (Table 1). As an example, the IEDB database (http://www.iedb.org/) collect a large number of peptides already identified, documented in literature, or voluntarily submitted by users. It includes peptides known as MHC binders derived from alloantigens and antigens involved in pathogen infections, allergies, and autoimmune diseases. The database can be easily accessed through a search engine retrieving information about host specificity, HLA restriction, and binding affinity. It also provides analysis and prediction tools that require only antigen primary sequence [28].

Another example of database comprising huge number of peptides characterized and available in the literature is SYFPEITHI (http://www.syfpeithi.de/), which includes as well algorithms calculating binding affinity of a query peptide to a specific MHC type [55, 56].

Other more specific databases are available to date, most notably the HIV-dedicated B- and T-cell epitope database (http://www.hiv.lanl.gov/). As the above-cited databases, besides a search engine that allows the user to look for HIV epitopes specific for CTL or helper T lymphocytes, this database includes a panel of different tools that offer different search options and permit to work with HLA sequences providing graphical distribution of the most frequently targeted regions. 

Selecting target HLAs is another crucial step in epitope-based vaccinology, as an effective preparation has to include protective epitopes capable of binding MHCs in the majority of individuals; the IMGT HLA database (http://www.ebi.ac.uk/ipd/imgt/hla/) provides updated information about HLA alleles and polymorphisms with their relative distribution among the population [57].

### 3.2. *In Silico* Approaches: Structure-Based Algorithms

Several algorithms are currently used in T-cell epitopes prediction. Considering the increasing importance of *in silico* modeling in predicting protein-protein interaction, here we review the MHC binding prediction tools. MHC-binding predictors can be divided in two main categories relying on structural or sequence analysis; being complex and computationally expensive, few structure-based algorithms are available to date. 

Structure-based MHC binding prediction methods can be clustered in three main categories, based on protein threading, homology modeling, or protein-protein docking. Protein-threading methods use a known peptide/MHC complex structure to predict binding features of others peptides to the same MHC; this process involves the substitution of the original peptide with the one to be tested followed by a side chains orientation optimization [58, 59]. Discrimination of binders from nonbinders is then performed using different scoring schemes.

Homology modeling has been used to predict MHC-binding peptides and potentially represents an improvement of threading methods since it allows to model both novel peptides and homologous MHC starting from a crystallographic structure [60, 61].

Docking techniques differ from protein threading and homology modeling since they do not rely on a template peptide; their aim is in fact to explore all possible query peptide orientations in the binding with MHCs. Many different docking-based approaches have been extensively used, either based on rigid docking evaluation or on molecular dynamics, and Monte Carlo simulations performed to find the best fitting geometry and evaluate binding strength [62, 63]. These techniques allowed to model proteins of unknown structures and, most importantly, to address experimental studies in the comprehension of protective antigen regions involved in the docking but are not suitable to complete antigenic mapping.

### 3.3. *In Silico* Approaches: Sequence-Based Algorithms

Sequence-based methods have been far more developed considering their low computational cost and independency from available crystallographic structures. As happened for B cell epitopes prediction algorithms, in the last decade these methods significantly improved and, starting from simple statistical sequence analysis, have moved towards machine-learning methods.

First attempts were based on the evidence that MHC binding pocket presents cavities with specific residues that require a certain degree of complementarity with specific epitope residues, defined as anchor residues; these algorithms thus search for this type of residues in specific positions, giving the highest contribute in MHC/epitope bindings. However, this strategy completely dismisses the contribute of nonanchor residues, resulting in a prediction lacking specificity and sensitivity [64].

From a simple search of specific residues, new algorithms moved towards a binding matrix-based strategy that takes into account residue frequencies at each epitope position; scoring matrices are built on the sequences of experimentally known binders and comprise information about position-specific frequencies and binding affinity. Binding matrices algorithms return more reliable results, and some of them, such as SYFPEITHI (http://www.syfpeithi.de/Scripts/MHCServer.dll/EpitopePrediction.htm) and BIMAS (http://www-bimas.cit.nih.gov/), are still used and are part of many prediction servers [56, 65]. An improvement of binding matrices algorithms is represented by the stabilized matrix method (SMM); Peters and Sette optimized a standard matrix algorithm strategy including a new score for heavy nonbinders peptides and a regularization technique to minimize the distance between predicted scores and experimental binding affinities contained in the training dataset [66]. The combination of this SMM with a pair coefficient that calculate a score for peptide residue pairs is included in the IEDB database and, together with ANN algorithms, showed the best prediction results in a broad comparative evaluation of MHC I binders predictors [67–69].

Novel algorithms evolved and adopted machine-learning approaches such as ANNs, HMMs, and SVMs; these algorithms have the advantage to perform predictions handling nonlinear data. ANN algorithms are some of the best predictors; they represent epitopes features as amino acid descriptors and perform complex pattern recognition after being trained with a dataset of epitopic and nonepitopic peptides. Their main drawback is the capability to predict epitopes only when query peptides and the training dataset are of the same length. Considering MHC II epitopes length variability, an alignment of peptides contained in the dataset to search for a pattern in the sequence core of defined length is necessary [70].

To date there are tens of online tools to predict MHC I and MHC II epitopes; considering the lack of standardization in dataset, the heterogeneity in output features and a highly variable performance of the same algorithm depending on the HLA type, defining the most reliable predictor, is not trivial. Lin et al. defined a standard benchmark protocol for both MHC I and MHC II predictors and tested the performance of the most used algorithms [68, 70]. The first conclusion describes a lower prediction accuracy (measured as *A*_ROC_) for MHC II algorithms than for MHC I that is explained by the increased biological complexity in terms of peptide length. Among the others, they identify the ANN and SMM algorithms embedded in the IEDB website together with NetMHC (http://www.cbs.dtu.dk/services/NetMHC/) ANN as the best predictors for MHC I epitopes [66, 71, 72]. For MHC II epitopes, the ANN algorithm Net-MHCIIpan (http://www.cbs.dtu.dk/services/NetMHCIIpan/), the SMM IEDB and PROPRED (http://www.imtech.res.in/raghava/propred/) outperformed the other methods [73, 74].

Although MHC binding prediction algorithms have reached high performances, they do not take into account the biological processes involved in epitopes production; predicted epitopes might not in fact be produced from antigen degradation [75, 76]. Many strategies exploiting sequence-based and machine-learning algorithms have been developed to predict antigen cleavage from the proteasome/immunoproteasome and TAP interactions. These tools are available either as stand-alone online servers or integrated with other algorithms to provide a complete prediction from the whole antigen to single epitopes. Furthermore, many of them are embedded in online databases.

Among the others, the ANN algorithm NetChop-3.0 (http://www.cbs.dtu.dk/services/NetChop/) seems to be the best predictor for proteasome cleavage; it is part of the online server NetCTL (http://www.cbs.dtu.dk/services/NetCTL/) for complete prediction [77, 78]. The whole suite is also part of the IEDB analysis tools. Another processing prediction algorithm is FragPredict, which predict both antigen cleavage searching and TAP binding; it uses a statistical analysis to search for amino acid motifs characterizing proteolytic sites [79, 80]. FragPredict is part of the MAPPP server (http://www.mpiib-berlin.mpg.de/MAPPP/), which takes positive peptides and further analyzes them for MHC binding through the BIMAS and SYFPEITHI algorithms [81] (Table 1).

### 3.4. *In Vitro* Approaches: Cell-Based Methods

Experimental techniques for T-cell epitopes mapping can be roughly divided in two main groups defined as cell based and cell free.

Cell-based techniques mainly involve the screening of synthetic peptides on T-cell population to evaluate binding specificity. The aforementioned computational methods play a fundamental role to focus the analysis on a selected cohort of peptides, reducing the number of potential ligands to be tested. Hereafter, we review the most common approaches used to date [82].

A broadly used cell-based approach is the enzyme linked immunospot assay (ELISPOT) [83]; it evaluates T-cell cytokines secretion levels (generally IFN-*γ*) after antigen recognition. In details, lymphocytes are incubated on plates coated with anticytokines Abs with different peptides to be tested. Produced cytokines are captured and secretory activity is then evaluated immunochemically. The advantages derived from this technique mainly consist in its high resolution (single-cell) and high throughput results that can be further improved by the use of dedicated scanners allowing the scaling-up of the technique.

Other cell-based assays are based on flow cytometry techniques that allow the selection of activated T cells. A widely used approach involves the culture of T cells in copresence of putative epitopes and a secretion inhibitor [84]. Activated cells are then sorted through after intracellular staining of retained cytokines with labeled Abs; different cytokines can be simultaneously evaluated using specific fluorescent-labeled antibodies. The most important limitation of this technique consists in the requirement of high quality sorting facilities. 

Lymphoproliferation assays rely as well on cytometric relevation; they consist in the uptake of the CFSE dye from T cells before activation [85]. After incubation with different peptides, antigen stimulation is evaluated through dye dilution caused by activated T-cell proliferation.

The use of cell-based techniques presents several advantages, most notably the possibility to test the putative T cell-activating peptides directly against target cells. The main drawback consists in the need to be addressed by preliminary computational studies to reduce time and resources expense.

### 3.5. *In Vitro* Approaches: Cell-Free Methods

Many cell-free methods have been developed to identify a definite antigen region potentially able to stimulate an effective T-cell response. Here, we briefly review one of the most promising approaches adopted in this research field [86]. It consists in recreating the antigen-processing compartment through the proteolytic digestion of an antigen of interest. The whole antigen is incubated with adequate soluble MHC molecules and proteases (mainly cathepsins and exopeptidases). Digested peptides specifically recognized by MHC molecules are bound and eluted after immunoprecipitating the complex, and T epitopes can then be analyzed by MS to identify immunogenic protein domains. The most important advantage of this assay relies on the direct employment of the whole antigen present on the pathogen to be targeted and on the simulation of its protelytic digestion into immunogenic peptides. The use of the entire antigen can permit, in fact, the identification of antigen-derived peptides that can be omitted during a synthetic peptide library design and/or during the *in silico* evaluation of the peptides to be assayed [87]. Moreover, the use of mass spectrometry methods allows the recognition of peptide posttranslational modifications that can affect the binding.

## 4. Discussion

Several approaches combining the use of computational analysis with laboratory techniques have been widely described in the scientific literature [88–93]. Here we take influenza virus as an example of hypervariable pathogen that requires the development of novel vaccinal strategies to elicit a broad immune response. Two studies are reported as examples of B-cell epitope characterization and T-cell-activating peptides identification through the combination of computational and experimental approaches.

First example regards the epitope characterization of PN-SIA28, a mAb endowed with potent neutralizing activity against highly phylogenetically divergent isolates of Influenza A virus and directed against a conserved region of the surface glycoprotein hemagglutinin. PN-SIA28 has been characterized through different experimental and *in silico* approaches [94–96]. In particular, Clementi et al. employed techniques such as random peptide library screening, alanine scanning on HA, and *in vitro *generation of escape viral variant under mAb selective pressure. The experimental derived data have been then analyzed through freely available bioinformatics tools, allowing the identification of the putative epitope recognized by PN-SIA 28. More in details, the analysis of mimotopes sequences selected through the peptide panning technique has been performed using *Pepitope,* a freely available online server. It allowed the identification of putative PN-SIA28 epitope through the superimposition of panning-selected peptide structural motifs on HA crystal structures. Epitope preliminary prediction has been confirmed and extended by experimental approaches such as alanine scanning.

As previously described, T-cell epitopes prediction requires the use of databases and bioinformatic tools to address experimental studies. Predictive algorithms are employed to significantly reduce the number of putative peptides to be tested against T cells. As an example, Wang et al. used the NetCTL server, which rely on ANN-based algorithms to predict proteasomal cleavage, interaction propensity to TAP and MHC bindings to obtain a limited number of putative HLA-binding peptides derived from influenza A proteins [97]. The binding-dependent T-cell activation of *in silico* identified peptides has been then evaluated through cell-based techniques such as ELISPOT and intracellular cytokines staining. This integrated study identified 13 peptides highly conserved among the H5N1 Influenza subtype able to elicit a T cells-mediated immune response. Later on, the same research group used an almost identical approach to extend their analysis to protein domains less conserved but more protective [98]. Considering both researches, Wang et al. characterized 30 peptides capable of elicit a cellular immune response that require *in vivo* studies to verify their protective activity. These combined approaches are largely used to target different hypervariable viruses [99, 100] and have been extensively used as well to study nonviral pathogens [101–104].

## 5. Conclusions

Hypervariable viruses still represent a major world health threat. The identification of conserved protein domains, shared among the different viruses and able to elicit a protective immune response, opens new perspectives in the development of epitope-based vaccines. In particular, the discovery of protective mAbs, able to target these broadly shared protein motifs, permits to work on the identification of peptides able to mimic these epitopes, and hopefully, to elicit an immune response similarly protective. Moreover, the possibility to identify peptides able to elicit an effective T-cell response against these viruses can enormously implement the efficacy of a new vaccine formulation able to elicit both T- and B-cell protective responses (Figure 1). Here, we reviewed different strategies based on experimental techniques and aimed to reach this main “goal” through the use of “*in silico*” strategies allowing to address and analyze the empirical obtained data and reducing experimental time and costs by improving identification efficacy.

## Figures and Tables

**Table 1:** Examples of the most commonly used databases and sequence-based algorithms for T-cell epitopes prediction.

Databases	Link	Algorithms used (cited ones)
Immune Epitope Database (IEDB)	http://www.iedb.org/	Stabilized Matrix Method-NetMHC-NetMHCIIpan-NetChop
SYFPEITHI	http://www.syfpeithi.de/	SYFPEITHI
HIV Molecular Immunology Database	http://www.hiv.lanl.gov/	
IMGT/HLA Database	http://www.ebi.ac.uk/ipd/imgt/hla/	

Sequence-based algorithms	Link	Brief description

SYFPEITHI	http://www.syfpeithi.de/Scripts/MHCServer.dll/EpitopePrediction.htm	Use of anchor residues Score based on frequency in natural ligands
BIMAS	http://www-bimas.cit.nih.gov/molbio/hla_bind/	MHC I epitopes predictor Use of coefficient tables of dissociation halftime
Stabilized Matrix Method	http://tools.immuneepitope.org/main/html/tcell_tools .html	*Peters and Sette, 2005* Score system for nonbinders Use of training datasets
NetMHC	http://cbs.dtu.dk/services/NetMHC/	Artificial neural network MHC I epitopes predictor Trained with 57 human HLA
NetMHCIIpan	http://cbs.dtu.dk/services/NetMHCIIpan/	Artificial neural network MHC II epitopes predictor Analyze >500 HLA-DR alleles
PROPRED	http://www.imtech.res.in/raghava/propred/	Use of quantitative matrices derived from the literature MHC II epitopes predictor
NetChop	http://cbs.dtu.dk/services/NetChop/	Artificial neural network Proteasome cleavage predictor Part of NetCTL server
FragPredict	http://www.mpiib-berlin.mpg.de/MAPPP/expertquery.html	Proteasomal cleavage sites and proteolytic fragments predictor Part of MAPPP server
